# A possible founder *IRAK4* variant in Okinawa, Japan

**DOI:** 10.70962/jhi.20260119

**Published:** 2026-09-15

**Authors:** Dan Tomomasa, Tomoko Uehara, Tomotada Takayama, Madoka Nishimura, Ryosuke Wakatsuki, Xi Yang, Satoshi Okada, Akihiro Fujimoto, Hirokazu Kanegane

**Affiliations:** 1 Department of Pediatrics and Developmental Biology, Institute of Science Tokyo, Tokyo, Japan; 2 Department of Pediatrics, Naha City Hospital, Okinawa, Japan; 3 General Pediatrics, Okinawa Prefectural Nanbu Medical Center & Children’s Medical Center, Okinawa, Japan; 4 Department of Pediatrics, Graduate School of Medical Sciences Kumamoto University, Kumamoto, Japan; 5 Ministry of Education Key Laboratory of Child Development and Disorders, Chongqing Key Laboratory of Child Infection and Immunity, National Clinical Research Center for Child Health and Disorders, Chongqing, China; 6 Division of Rheumatology and Immunology, Children’s Hospital of Chongqing Medical University, Chongqing, China; 7 Department of Pediatrics, Hiroshima University Graduate School of Biomedical and Health Sciences, Hiroshima, Japan; 8 Department of Human Genetics, School of International Health, Graduate School of Medicine, The University of Tokyo, Tokyo, Japan; 9 Department of Child Health and Development, Institute of Science Tokyo, Tokyo, Japan

## Abstract

A cluster of cases of IRAK4 deficiency has been identified in Okinawa Prefecture, Japan. All affected families carry the homozygous c.123_124insA variant, suggesting a founder effect and supporting the implementation of targeted newborn screening of IRAK4 deficiency in Okinawa.

## Teaser

Tomomasa et al. describe that a clustering of patients with IRAK4 deficiency was identified in Okinawa Prefecture, Japan. All affected families carried the homozygous c.123_124insA variant, which suggests a founder effect and supports the implementation of newborn screening for IRAK4 deficiency in Okinawa Prefecture.

IRAK4 deficiency is a rare autosomal recessive inborn error of immunity caused by biallelic pathogenic variants in *IRAK4*. Patients with IRAK4 deficiency are characterized by an impaired innate immune response and a predisposition to invasive bacterial infections, including pneumococcal meningitis. Although the c.123_124insA variant has been reported in multiple Japanese patients with IRAK4 deficiency, its geographical distribution and population genetic characteristics in East Asia remain unclear (1). To investigate this issue, we conducted a comprehensive review of the published literature on IRAK4 deficiency.

**Figure 1.**
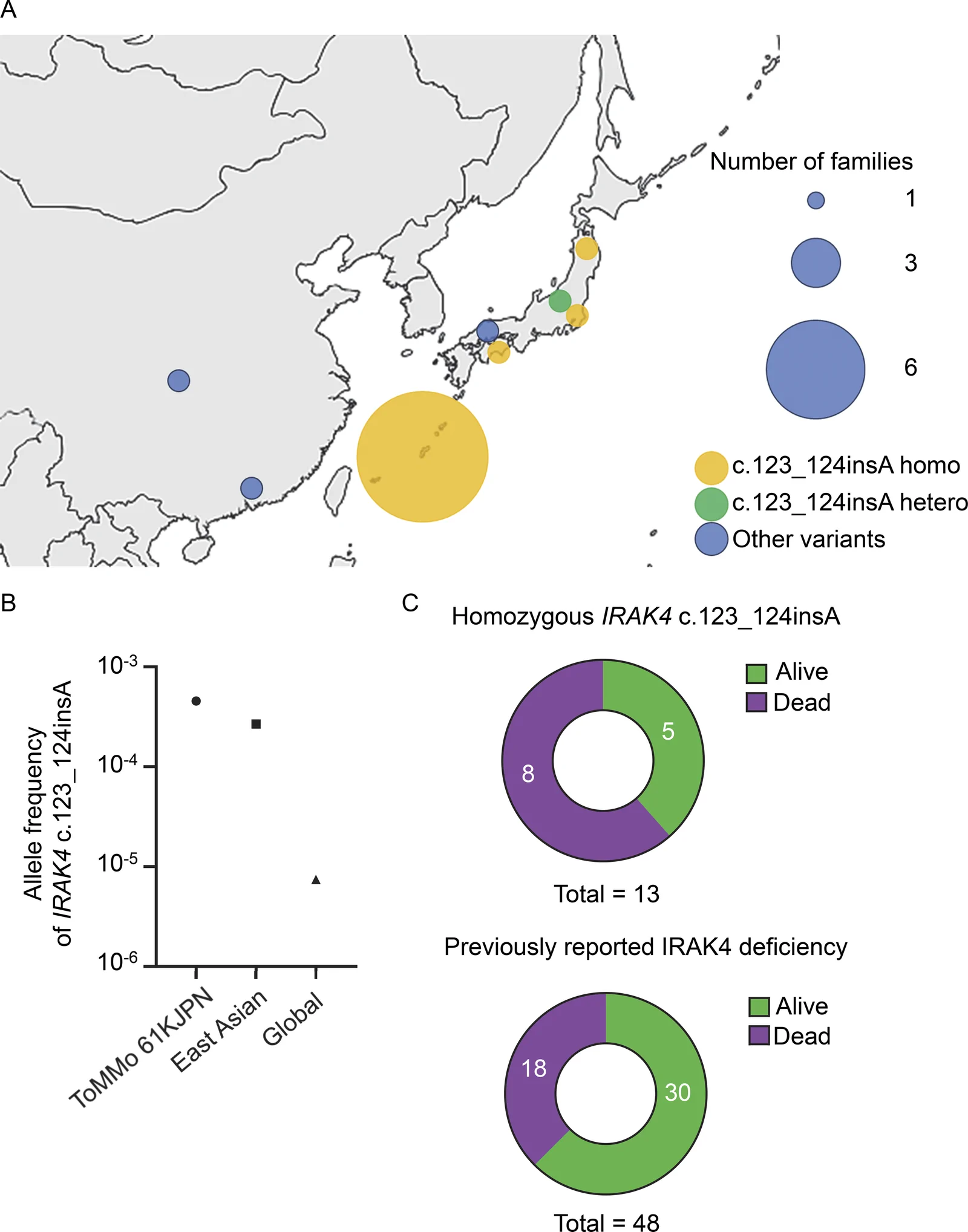
**Geographic distribution, allele frequency, and clinical features of IRAK4 deficiency. (A)** Geographic distribution of reported families with IRAK4 deficiency in East Asia. Families were identified from previously published reports (Picard et al., 2010; Takada et al., 2016; Yoshikawa et al., 2007; Shichijo et al., 2015; Uehara et al., 2021; Nishimura et al., 2020; Kanno et al., 2021; Shimabukuro et al., 2023; Duan et al., 2022; Liu et al., 2021; Luo et al., 2021; Peng et al., 2025; Zhang et al., 2024; Shimabukuro et al., 2010, *Nihon Shonika Gakkai Zasshi*, 114:583 [in Japanese]; Kitani et al., 2018, *Kanagawa Igakukai Zasshi*, 45:216-217 [in Japanese]). **(B)** Allele frequency of the *IRAK4* c.123_124insA variant across ToMMo 61KJPN, East Asian, and global populations. **(C)** Mortality was 62% (8/13) among patients homozygous for c.123_124insA compared with 38% (18/48) in the previously reported cohort. ToMMo, Tohoku Medical Megabank Organization.

The distribution of families with IRAK4 deficiency was highly concentrated in Okinawa Prefecture (Fig. 1 A). Given that Okinawa accounts for only ∼1% of the Japanese population, the concentration of affected families in this region is striking. Notably, all six families carried the same homozygous *IRAK4* c.123_124insA variant. In the Genome Aggregation Database (https://gnomad.broadinstitute.org), this variant was only found in East Asia with an allele frequency of 0.0002677 (Fig. 1 B). According to the database of the Tohoku Medical Megabank Organization (https://www.megabank.tohoku.ac.jp/), the allele frequency of this variant is 0.000457 out of 122,426 Japanese alleles. These observations raise the possibility that the *IRAK4* c.123_124insA variant represents a founder variant in the Okinawan population. Of the 13 affected individuals, 9 (69%) developed symptoms before 1 year of age, 8 (62%) had meningitis, and 8 (62%) had invasive *Streptococcus pneumoniae* infection (Table 1). Notably, mortality among patients homozygous for c.123_124insA was 62% (8/13), compared with 38% in the largest previously reported cohort of IRAK4 deficiency (2) (Fig. 1 C).

**Table 1.** Characteristics of IRAK4-deficiency patients with homozygous c.123_124insA variants

​	Age at onset	Major infections	Pathogens	Outcome	Region
P1	2 wk	Urachal infection, meningitis, hip arthritis	MRSA, *S. pneumoniae*	Deceased	Tohoku
P2	-	None	None	Alive	Tohoku
P3	-	None	None	Alive	Tohoku
P4	6 mo	Subcutaneous abscess, meningitis	*S. aureus*, *S. pneumoniae*	Deceased	Shikoku
P5	7 mo	Intra-abdominal abscess, liver abscess, meningitis	*P. aeruginosa*, *S. pneumoniae*	Deceased	Okinawa
P6	9 mo	Necrotizing fasciitis, sepsis	*P. aeruginosa*	Deceased	Okinawa
P7	1 mo	Omphalitis, subcutaneous abscess, meningitis	*S. aureus*, GBS, *S. pneumoniae*	Alive	Okinawa
P8	8 mo	Meningitis, sepsis	*S. pneumoniae*	Deceased	Okinawa
P9	3 mo	Meningitis, sepsis	*S. pneumoniae*	Alive	Okinawa
P10	3 years 10 mo	Osteomyelitis, meningitis	*S. pneumoniae*	Alive	Kanto
P11	10 mo	Sepsis	*S. pneumoniae*	Deceased	Kanto
P12	1 year	Necrotizing fasciitis, sepsis	*P. aeruginosa*	Deceased	Okinawa
P13	9 mo	Ileocecitis, sepsis, meningitis	*P. aeruginosa*	Deceased	Okinawa

MRSA, methicillin-resistant *S*.* aureus*; *S. aureus*, *Staphylococcus aureus*; *P. aeruginosa*, *Pseudomonas aeruginosa*; GBS, group B *Streptococcus*.

Founder variants introduced into Japan from neighboring East Asian populations, possibly through migration from northern China via the Korean Peninsula, have previously been reported (4). However, the distribution of the *IRAK4* c.123_124insA variant appears to differ from this pattern. To our knowledge, no cases of IRAK4 deficiency have been reported from Korea. Although the *IRAK4* c.123_124insA variant is present in Japanese populations, it has not been identified in reported Chinese patients with IRAK4 deficiency. In contrast, the c.540delT variant was detected in three of the five reported Chinese patients and is present in the Chinese population (allele frequency of 0.0001667) according to the NyuWa genome resource (http://bigdata.ibp.ac.cn/NyuWa/), suggesting a distinct geographical distribution of pathogenic *IRAK4* variants in East Asia. These findings further support the possibility that the c.123_124insA variant became enriched in the Okinawan population through a founder effect. Okinawa was historically relatively isolated from mainland Japan during the Ryukyu Kingdom period (1429–1879), which may have contributed to reduced gene flow and the persistence of founder variants within the local population. Moreover, its relatively small population size may have amplified the effects of genetic drift, leading to an increased frequency of deleterious founder alleles. The *SCN5A* p.E1784K variant associated with long QT syndrome has also been reported to suggest a founder effect in the Okinawan population (5). However, because we did not perform haplotype analysis, we cannot exclude the possibility that the c.123_124insA variant represents a mutational hotspot rather than a founder variant. The c.123_124insA variant has been shown to result in complete loss of IRAK4 protein expression. Furthermore, Takada et al. (1) previously demonstrated complete loss of lipopolysaccharide-induced tumor necrosis factor-α (TNF-α) production by CD14^+^ monocytes in a patient with homozygous c.123_124insA variants. Although the clinical phenotype was heterogeneous, patients homozygous for the variant frequently presented in infancy and exhibited a high mortality rate, consistent with the severe functional consequences of this variant. Delayed umbilical cord separation has been reported in patients with IRAK4 deficiency (1). Given the apparent enrichment of this variant in Okinawa and the substantial burden of severe disease observed among affected individuals, early identification of affected infants may be particularly important in this region. The potential benefit of early diagnosis is illustrated by two patients with homozygous c.123_124insA variants, who were identified in early infancy because of a positive family history; early prophylaxis prevented invasive disease (1). In Okinawa, a pilot newborn screening program has been initiated to evaluate the utility of an LPS-induced TNF-α production assay in infants with umbilical cord separation delayed beyond 2 wk after birth (3). To date, more than 500 infants have been tested, but no positive cases have been identified. Eight tertiary care centers and four general clinics have participated in the program. While functional screening assays may facilitate case detection, the presence of a recurrent founder variant also suggests a potential role for targeted genetic testing as a rapid diagnostic strategy in this region.

In conclusion, the geographical clustering of affected families and the shared homozygous *IRAK4* c.123_124insA variant are consistent with a founder effect in Okinawa, which may contribute to the increased incidence of IRAK4 deficiency in this population.

## Ethics approval

Genetic analysis was performed after obtaining written informed consent from the patients. This study was performed in accordance with the Helsinki declaration and approved by the Ethics Committee of Institute of Science Tokyo (approval number: G2019-004).

## Consent to participate

Informed consent was obtained from all patients included in this study and their parents.

## Consent for publication

Informed consent was obtained from all participants or their parents.

## Data Availability

The datasets used in this study are not publicly available to protect participant/patient anonymity. Requests to access the datasets can be made to the corresponding author.
